# Tris(ethane-1,2-diamine)copper(II) bis­(trifluoro­acetate)

**DOI:** 10.1107/S1600536808001438

**Published:** 2008-01-18

**Authors:** Elena V. Karpova, Maxim A. Zakharov, Alexandr I. Boltalin, Victor B. Rybakov

**Affiliations:** a Inorganic Chemistry Division, Chemistry Department, Moscow State University, 119991 Leninskie Gory 1-3, Moscow, Russian Federation; b General Chemistry Division, Chemistry Department, Moscow State University, 119991 Leninskie Gory 1-3, Moscow, Russian Federation

## Abstract

In the title complex, [Cu(H_2_NCH_2_CH_2_NH_2_)_3_](CF_3_COO)_2_, the environment of the Cu atom is distorted octa­hedral, formed by six N atoms from three chelating ethane-1,2-diamine ligands. The Cu—N distances range from 2.050 (2) to 2.300 (2) Å. This complex cation and the two trifluoro­acetate anions are connected by weak N—H⋯O and N—H⋯F hydrogen bonds, forming a three-dimensional framework. In both anions, the F atoms are disordered over two positions; in one the site-occupancy factors are 0.55 and 0.45, in the other the values are 0.69 and 0.31.

## Related literature

For other carboxyl­ate complexes, see: Karpova *et al.* (1998 ▶); Karpova *et al.* (2001 ▶); Gutnikov *et al.* (2006 ▶); Karpova *et al.* (2007 ▶).
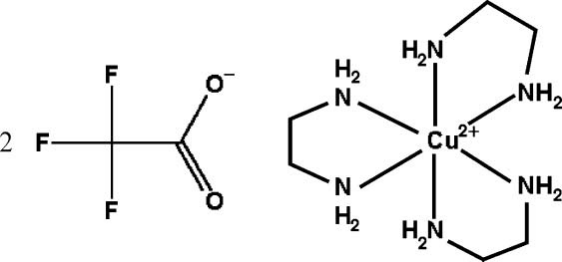


## Experimental

### 

#### Crystal data

[Cu(C_2_H_8_N_2_)_3_](C_2_F_3_O_2_)_2_
*M* *_r_* = 469.90
Triclinic, 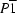

*a* = 8.582 (6) Å
*b* = 9.316 (6) Å
*c* = 12.859 (7) Å
α = 74.73 (3)°
β = 84.69 (4)°
γ = 69.56 (3)°
*V* = 929.3 (10) Å^3^
*Z* = 2
Mo *K*α radiation
μ = 1.26 mm^−1^
*T* = 293 (2) K
0.15 × 0.1 × 0.08 mm

#### Data collection

Bruker SMART CCD area-detector diffractometer
Absorption correction: none
5406 independent reflections
4277 reflections with *I* > 2σ(*I*)

#### Refinement

*R*[*F* ^2^ > 2σ(*F* ^2^)] = 0.039
*wR*(*F* ^2^) = 0.092
*S* = 0.94
5406 reflections
300 parameters
45 restraints
H-atom parameters constrained
Δρ_max_ = 0.24 e Å^−3^
Δρ_min_ = −0.24 e Å^−3^

### 

Data collection: *SMART* (Bruker, 2000 ▶); cell refinement: *SAINT* (Bruker, 2000 ▶); data reduction: *SAINT*; program(s) used to solve structure: *SHELXS97* (Sheldrick, 2008 ▶); program(s) used to refine structure: *SHELXL97*; molecular graphics: *ORTEP-3* (Farrugia, 1997 ▶); software used to prepare material for publication: *WinGX* (Farrugia, 1999 ▶).

## Supplementary Material

Crystal structure: contains datablocks global, I, publication_text. DOI: 10.1107/S1600536808001438/wn2235sup1.cif

Structure factors: contains datablocks I. DOI: 10.1107/S1600536808001438/wn2235Isup2.hkl

Additional supplementary materials:  crystallographic information; 3D view; checkCIF report

## Figures and Tables

**Table 1:** Hydrogen-bond geometry (Å, °)

*D*—H⋯*A*	*D*—H	H⋯*A*	*D*⋯*A*	*D*—H⋯*A*
N2—H2*B*⋯F1*A*	0.90	2.54	3.248 (17)	136
N2—H2*B*⋯F3*B*	0.90	2.55	3.41 (2)	161
N6—H6*B*⋯O1	0.90	2.15	3.048 (3)	179
N1—H1*A*⋯O2^i^	0.90	2.35	3.141 (3)	147
N1—H1*A*⋯O1^i^	0.90	2.53	3.377 (4)	156
N4—H4*A*⋯O2^i^	0.90	2.34	3.175 (3)	154
N6—H6*A*⋯O1^i^	0.90	2.56	3.326 (3)	143
N1—H1*B*⋯O2^ii^	0.90	2.11	3.002 (3)	173
N2—H2*A*⋯F4*A*^ii^	0.90	2.47	3.267 (15)	148
N3—H3*A*⋯O3^ii^	0.90	2.09	2.958 (3)	162
N5—H5*A*⋯O3^ii^	0.90	2.36	3.126 (3)	143
N2—H2*A*⋯O4^iii^	0.90	2.41	3.037 (3)	127
N4—H4*B*⋯O4^iv^	0.90	2.11	2.997 (3)	168
N5—H5*B*⋯O3^iv^	0.90	2.13	3.013 (3)	167

Symmetry codes: (i) 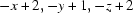; (ii) 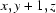; (iii) 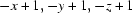; (iv) 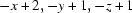.

**Fig. 1.**
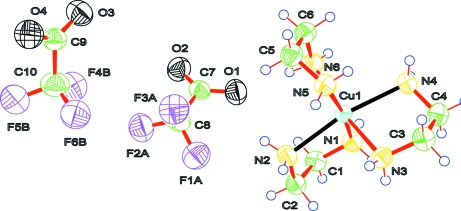
A view of the title compound, with the atom numbering scheme. Displacment ellipsoids are shown at the 50% probability level. H atoms are drawn as small spheres of arbitrary radius. Only the major disorder components are shown.

## supplementary crystallographic information

### Comment

The present investigation is a continuation of experimental work to study the structure and properties of different carboxylate complexes (Karpova *et al.*, 1998; Karpova *et al.*, 2001; Gutnikov *et al.*, 2006; Karpova *et al.*, 2007). In the title compound, Cu(H_2_NCH_2_CH_2_NH_2_)_3_.(CF_3_COO)_2_, the asymmetric unit consists of a complex cation and two crystallographically independent anions. The environment of the Cu atom is distorted octahedral, formed by six N atoms from three chelate ethylenediamine groups. The two trifluoroacetate anions form N—H···O and N—H···F hydrogen bonds with NH_2_ groups of the cation, forming a three-dimensional framework.

### Experimental

An ethanol solution of [Cu(CF_3_COO)_2_(CH_3_CN)]_2_(CH_3_CN)_2_ was mixed with ethylenediamine in a 1:4 molar ratio. After several days blue prism-shaped crystals were formed in a desiccator over P_4_O_10_.

### Refinement

All H atoms were positioned geometrically and refined using a riding model (including free rotation about C—C or C—N bonds) with C—H = 0.97 Å, N—H = 0.90 Å and with *U*_iso_(H) = 1.2*U*_eq_(C,*N*). The DELU option in *SHELXL97* was used with parameters 0.0001, 0.0001 for all bonds Cu1—N; for all C—O and C—F bonds. The MERG option with parameter 2 was used before refinement. The F atoms are disordered over two positions, with site occupancy ratios 0.55 (3)/0.45 (3) and 0.69 (2)/0.31 (2).

### Crystal data

**Table:**

[Cu(C_2_H_8_N_2_)_3_](C_2_F_3_O_2_)_2_	*Z* = 2
*M**_r_* = 469.90	*F*_000_ = 482
Triclinic, *P*1	*D*_x_ = 1.679 Mg m^−^^3^
Hall symbol: -P 1	Mo *K*α radiation λ = 0.71073 Å
*a* = 8.582 (6) Å	Cell parameters from 250 reflections
*b* = 9.316 (6) Å	θ = 18–24º
*c* = 12.859 (7) Å	µ = 1.26 mm^−^^1^
α = 74.73 (3)º	*T* = 293 (2) K
β = 84.69 (4)º	Prism, blue
γ = 69.56 (3)º	0.15 × 0.1 × 0.08 mm
*V* = 929.3 (10) Å^3^	

### Data collection

**Table:**

Bruker SMART CCD area-detector diffractometer	5406 independent reflections
Radiation source: fine–focus sealed tube	4277 reflections with *I* > 2σ(*I*)
Monochromator: graphite	θ_max_ = 30.0º
Detector resolution: 0.1 pixels mm^-1^	θ_min_ = 1.6º
*T* = 293(2) K	*h* = −11→12
φ and ω scans	*k* = 0→13
Absorption correction: none	*l* = −17→18

### Refinement

**Table:**

Refinement on *F*^2^	Secondary atom site location: difference Fourier map
Least-squares matrix: full	Hydrogen site location: inferred from neighbouring sites
*R*[*F*^2^ > 2σ(*F*^2^)] = 0.039	H-atom parameters constrained
*wR*(*F*^2^) = 0.092	*w* = 1/[σ^2^(*F*_o_^2^) + (0.0567*P*)^2^] where *P* = (*F*_o_^2^ + 2*F*_c_^2^)/3
*S* = 0.94	(Δ/σ)_max_ = 0.001
5406 reflections	Δρ_max_ = 0.24 e Å^−^^3^
300 parameters	Δρ_min_ = −0.24 e Å^−^^3^
45 restraints	Extinction correction: none
Primary atom site location: structure-invariant direct methods	

### Special details

**Table:**

Experimental. The original *HKL* file was deleted and the present study was conducted using the merged data set.
Geometry. All s.u.'s (except the s.u.'s in the dihedral angle between two l.s. planes) are estimated using the full covariance matrix. The cell s.u.'s are taken into account individually in the estimation of s.u.'s in distances, angles and torsion angles; correlations between s.u.'s in cell parameters are only used when they are defined by crystal symmetry. An approximate (isotropic) treatment of cell s.u.'s is used for estimating s.u.'s involving l.s. planes.
Refinement. Refinement of *F*^2^ against ALL reflections. The weighted *R*–factor *wR* and goodness of fit *S* are based on *F*^2^, conventional *R*–factors *R* are based on *F*, with *F* set to zero for negative *F*^2^. The threshold expression of *F*^2^ > 2σ(*F*^2^) is used only for calculating *R*–factors(gt) *etc*. and is not relevant to the choice of reflections for refinement. *R*–factors based on *F*^2^ are statistically about twice as large as those based on *F*, and *R*–factors based on ALL data will be even larger.

### Fractional atomic coordinates and isotropic or equivalent isotropic displacement parameters (Å^2^)

**Table:**

	*x*	*y*	*z*	*U*_iso_*/*U*_eq_	Occ. (<1)
Cu1	0.94303 (3)	0.85236 (3)	0.778785 (18)	0.03254 (8)	
N1	0.8821 (2)	0.8734 (2)	0.93404 (13)	0.0444 (3)	
H1A	0.9694	0.8131	0.9777	0.053*	
H1B	0.8587	0.9745	0.9362	0.053*	
C1	0.7376 (3)	0.8237 (3)	0.97256 (17)	0.0486 (5)	
H1C	0.6921	0.8610	1.0363	0.058*	
H1D	0.7726	0.7092	0.9923	0.058*	
C2	0.6060 (3)	0.8885 (3)	0.88718 (19)	0.0503 (5)	
H2C	0.5139	0.8505	0.9121	0.060*	
H2D	0.5641	1.0031	0.8708	0.060*	
N2	0.6812 (2)	0.8352 (2)	0.79038 (16)	0.0495 (4)	
H2A	0.6212	0.8977	0.7314	0.059*	
H2B	0.6866	0.7350	0.7968	0.059*	
N3	0.8604 (2)	1.1007 (2)	0.71134 (15)	0.0471 (4)	
H3A	0.8004	1.1244	0.6515	0.057*	
H3B	0.7948	1.1505	0.7589	0.057*	
C3	1.0036 (3)	1.1543 (3)	0.68450 (19)	0.0539 (5)	
H3C	0.9672	1.2681	0.6739	0.065*	
H3D	1.0531	1.1292	0.6177	0.065*	
C4	1.1308 (3)	1.0764 (3)	0.77268 (19)	0.0510 (5)	
H4C	1.2266	1.1102	0.7523	0.061*	
H4D	1.0843	1.1079	0.8382	0.061*	
N4	1.1826 (2)	0.9040 (2)	0.79228 (15)	0.0457 (4)	
H4A	1.2277	0.8564	0.8583	0.055*	
H4B	1.2577	0.8695	0.7428	0.055*	
N5	0.9835 (2)	0.8096 (2)	0.62842 (13)	0.0453 (4)	
H5A	0.8923	0.8658	0.5873	0.054*	
H5B	1.0688	0.8396	0.5965	0.054*	
C5	1.0217 (3)	0.6405 (3)	0.6384 (2)	0.0540 (5)	
H5C	1.0712	0.6134	0.5721	0.065*	
H5D	0.9208	0.6139	0.6522	0.065*	
C6	1.1414 (3)	0.5510 (3)	0.7304 (2)	0.0541 (5)	
H6C	1.1649	0.4382	0.7420	0.065*	
H6D	1.2451	0.5721	0.7144	0.065*	
N6	1.0652 (2)	0.6024 (2)	0.82682 (15)	0.0482 (4)	
H6A	1.1435	0.5783	0.8766	0.058*	
H6B	0.9914	0.5539	0.8555	0.058*	
C7	0.7420 (3)	0.3504 (3)	0.91576 (18)	0.0494 (4)	
C8	0.5826 (4)	0.4304 (4)	0.8472 (3)	0.0722 (6)	
C9	0.5876 (3)	0.1740 (3)	0.4611 (2)	0.0526 (5)	
C10	0.4415 (4)	0.2533 (4)	0.5269 (3)	0.0749 (6)	
O1	0.8137 (3)	0.4407 (2)	0.92515 (17)	0.0722 (5)	
O2	0.7776 (3)	0.2083 (2)	0.95315 (17)	0.0721 (5)	
O3	0.7270 (2)	0.1248 (2)	0.50104 (16)	0.0693 (5)	
O4	0.5502 (3)	0.1706 (3)	0.37227 (17)	0.0780 (6)	
F1A	0.495 (2)	0.5750 (14)	0.8639 (12)	0.087 (2)	0.55 (3)
F2A	0.4769 (16)	0.3531 (13)	0.8668 (13)	0.089 (3)	0.55 (3)
F3A	0.6064 (18)	0.4591 (14)	0.7404 (6)	0.093 (3)	0.55 (3)
F1B	0.471 (2)	0.537 (2)	0.8865 (14)	0.083 (2)	0.45 (3)
F2B	0.5182 (17)	0.3267 (11)	0.8290 (14)	0.088 (2)	0.45 (3)
F3B	0.6454 (13)	0.4960 (16)	0.7584 (10)	0.084 (3)	0.45 (3)
F4A	0.4601 (19)	0.166 (3)	0.6294 (12)	0.095 (4)	0.31 (2)
F5A	0.2886 (15)	0.281 (3)	0.4998 (12)	0.090 (4)	0.31 (2)
F6A	0.460 (3)	0.3837 (19)	0.529 (2)	0.110 (5)	0.31 (2)
F4B	0.4729 (10)	0.2254 (9)	0.6304 (5)	0.0850 (12)	0.69 (2)
F5B	0.3116 (8)	0.2068 (10)	0.5228 (6)	0.0872 (13)	0.69 (2)
F6B	0.3834 (11)	0.4109 (4)	0.4916 (4)	0.0858 (16)	0.69 (2)

### Atomic displacement parameters (Å^2^)

**Table:**

	*U*^11^	*U*^22^	*U*^33^	*U*^12^	*U*^13^	*U*^23^
Cu1	0.03390 (11)	0.03383 (12)	0.03075 (11)	−0.01198 (8)	−0.00040 (7)	−0.00858 (8)
N1	0.0453 (9)	0.0514 (10)	0.0380 (6)	−0.0172 (8)	0.0030 (6)	−0.0134 (6)
C1	0.0495 (12)	0.0522 (12)	0.0418 (11)	−0.0159 (10)	0.0017 (9)	−0.0102 (9)
C2	0.0459 (11)	0.0535 (12)	0.0534 (12)	−0.0178 (10)	−0.0004 (9)	−0.0151 (10)
N2	0.0431 (7)	0.0582 (10)	0.0527 (10)	−0.0214 (8)	0.0005 (7)	−0.0173 (9)
N3	0.0516 (9)	0.0419 (7)	0.0458 (9)	−0.0141 (6)	−0.0022 (7)	−0.0090 (6)
C3	0.0558 (13)	0.0551 (13)	0.0501 (12)	−0.0207 (11)	−0.0030 (10)	−0.0082 (10)
C4	0.0567 (13)	0.0513 (12)	0.0484 (12)	−0.0217 (10)	−0.0007 (10)	−0.0126 (10)
N4	0.0435 (7)	0.0539 (10)	0.0436 (9)	−0.0214 (7)	0.0008 (6)	−0.0122 (8)
N5	0.0497 (9)	0.0504 (9)	0.0371 (7)	−0.0169 (8)	−0.0005 (6)	−0.0130 (7)
C5	0.0562 (13)	0.0536 (13)	0.0544 (13)	−0.0187 (10)	−0.0003 (10)	−0.0168 (11)
C6	0.0556 (13)	0.0511 (12)	0.0564 (13)	−0.0160 (10)	0.0011 (10)	−0.0178 (11)
N6	0.0492 (10)	0.0468 (10)	0.0468 (10)	−0.0151 (8)	−0.0046 (8)	−0.0085 (8)
C7	0.0437 (11)	0.0599 (10)	0.0441 (11)	−0.0142 (9)	0.0010 (8)	−0.0166 (9)
C8	0.0540 (14)	0.0761 (15)	0.0748 (13)	−0.0134 (9)	−0.0125 (11)	−0.0069 (13)
C9	0.0515 (10)	0.0506 (12)	0.0544 (11)	−0.0160 (10)	0.0046 (9)	−0.0141 (10)
C10	0.0646 (14)	0.0808 (12)	0.0766 (12)	−0.0185 (13)	0.0130 (13)	−0.0279 (14)
O1	0.0695 (12)	0.0765 (11)	0.0789 (13)	−0.0323 (10)	−0.0057 (10)	−0.0208 (10)
O2	0.0746 (12)	0.0622 (9)	0.0781 (13)	−0.0197 (9)	−0.0112 (10)	−0.0157 (9)
O3	0.0593 (10)	0.0788 (13)	0.0688 (11)	−0.0210 (9)	−0.0023 (8)	−0.0191 (10)
O4	0.0763 (13)	0.0897 (15)	0.0685 (11)	−0.0219 (11)	−0.0079 (9)	−0.0262 (11)
F1A	0.094 (5)	0.078 (3)	0.086 (5)	−0.026 (2)	−0.003 (3)	−0.018 (4)
F2A	0.085 (4)	0.089 (3)	0.097 (5)	−0.036 (3)	−0.012 (3)	−0.018 (3)
F3A	0.099 (5)	0.099 (4)	0.0789 (16)	−0.032 (3)	−0.003 (2)	−0.0216 (19)
F1B	0.077 (4)	0.089 (6)	0.085 (6)	−0.026 (4)	0.004 (3)	−0.025 (5)
F2B	0.086 (5)	0.093 (3)	0.089 (6)	−0.033 (3)	0.006 (4)	−0.029 (3)
F3B	0.078 (4)	0.102 (5)	0.075 (3)	−0.033 (3)	0.001 (2)	−0.022 (3)
F4A	0.070 (4)	0.113 (10)	0.093 (4)	−0.038 (6)	0.005 (3)	0.000 (6)
F5A	0.078 (3)	0.116 (11)	0.083 (5)	−0.038 (6)	0.005 (3)	−0.029 (6)
F6A	0.097 (9)	0.098 (5)	0.144 (12)	−0.056 (6)	0.016 (8)	−0.020 (7)
F4B	0.085 (3)	0.091 (3)	0.0800 (15)	−0.033 (2)	0.0010 (16)	−0.0179 (17)
F5B	0.080 (2)	0.091 (3)	0.090 (3)	−0.030 (3)	−0.0023 (18)	−0.020 (3)
F6B	0.082 (4)	0.0829 (12)	0.090 (2)	−0.0239 (16)	−0.0062 (19)	−0.0192 (13)

### Geometric parameters (Å, °)

**Table:**

Cu1—N5	2.050 (2)	N5—H5A	0.9000
Cu1—N1	2.059 (2)	N5—H5B	0.9000
Cu1—N3	2.126 (2)	C5—C6	1.503 (3)
Cu1—N6	2.136 (2)	C5—H5C	0.9700
Cu1—N2	2.297 (2)	C5—H5D	0.9700
Cu1—N4	2.300 (2)	C6—N6	1.462 (3)
N1—C1	1.471 (3)	C6—H6C	0.9700
N1—H1A	0.9000	C6—H6D	0.9700
N1—H1B	0.9000	N6—H6A	0.9000
C1—C2	1.500 (3)	N6—H6B	0.9000
C1—H1C	0.9700	C7—O2	1.219 (3)
C1—H1D	0.9700	C7—O1	1.237 (3)
C2—N2	1.471 (3)	C7—C8	1.539 (4)
C2—H2C	0.9700	C8—F1B	1.291 (11)
C2—H2D	0.9700	C8—F3B	1.311 (7)
N2—H2A	0.9000	C8—F2A	1.312 (7)
N2—H2B	0.9000	C8—F3A	1.338 (7)
N3—C3	1.462 (3)	C8—F2B	1.345 (9)
N3—H3A	0.9000	C8—F1A	1.357 (9)
N3—H3B	0.9000	C9—O4	1.225 (3)
C3—C4	1.499 (3)	C9—O3	1.230 (3)
C3—H3C	0.9700	C9—C10	1.524 (4)
C3—H3D	0.9700	C10—F6A	1.286 (10)
C4—N4	1.466 (3)	C10—F5A	1.308 (11)
C4—H4C	0.9700	C10—F4B	1.322 (6)
C4—H4D	0.9700	C10—F5B	1.338 (5)
N4—H4A	0.9000	C10—F6B	1.338 (5)
N4—H4B	0.9000	C10—F4A	1.343 (11)
N5—C5	1.464 (3)		
			
N5—Cu1—N1	171.52 (7)	H5A—N5—H5B	108.3
N5—Cu1—N3	91.32 (8)	N5—C5—C6	107.7 (2)
N1—Cu1—N3	93.88 (8)	N5—C5—H5C	110.2
N5—Cu1—N6	81.82 (8)	C6—C5—H5C	110.2
N1—Cu1—N6	93.95 (8)	N5—C5—H5D	110.2
N3—Cu1—N6	169.22 (7)	C6—C5—H5D	110.2
N5—Cu1—N2	92.91 (8)	H5C—C5—H5D	108.5
N1—Cu1—N2	80.03 (8)	N6—C6—C5	108.1 (2)
N3—Cu1—N2	94.49 (8)	N6—C6—H6C	110.1
N6—Cu1—N2	94.15 (9)	C5—C6—H6C	110.1
N5—Cu1—N4	98.13 (8)	N6—C6—H6D	110.1
N1—Cu1—N4	89.38 (8)	C5—C6—H6D	110.1
N3—Cu1—N4	79.81 (8)	H6C—C6—H6D	108.4
N6—Cu1—N4	92.88 (8)	C6—N6—Cu1	107.29 (14)
N2—Cu1—N4	167.65 (7)	C6—N6—H6A	110.3
C1—N1—Cu1	110.81 (14)	Cu1—N6—H6A	110.3
C1—N1—H1A	109.5	C6—N6—H6B	110.3
Cu1—N1—H1A	109.5	Cu1—N6—H6B	110.3
C1—N1—H1B	109.5	H6A—N6—H6B	108.5
Cu1—N1—H1B	109.5	O2—C7—O1	129.9 (2)
H1A—N1—H1B	108.1	O2—C7—C8	115.2 (2)
N1—C1—C2	110.86 (18)	O1—C7—C8	114.9 (2)
N1—C1—H1C	109.5	F1B—C8—F3B	110.6 (7)
C2—C1—H1C	109.5	F1B—C8—F2A	86.2 (6)
N1—C1—H1D	109.5	F3B—C8—F2A	133.3 (5)
C2—C1—H1D	109.5	F1B—C8—F3A	118.9 (10)
H1C—C1—H1D	108.1	F3B—C8—F3A	28.3 (3)
N2—C2—C1	107.94 (19)	F2A—C8—F3A	105.2 (5)
N2—C2—H2C	110.1	F1B—C8—F2B	110.6 (7)
C1—C2—H2C	110.1	F3B—C8—F2B	110.6 (5)
N2—C2—H2D	110.1	F2A—C8—F2B	27.2 (3)
C1—C2—H2D	110.1	F3A—C8—F2B	82.7 (5)
H2C—C2—H2D	108.4	F1B—C8—F1A	21.0 (6)
C2—N2—Cu1	105.49 (14)	F3B—C8—F1A	90.5 (6)
C2—N2—H2A	110.6	F2A—C8—F1A	104.9 (5)
Cu1—N2—H2A	110.6	F3A—C8—F1A	103.9 (6)
C2—N2—H2B	110.6	F2B—C8—F1A	126.1 (7)
Cu1—N2—H2B	110.6	F1B—C8—C7	112.6 (9)
H2A—N2—H2B	108.8	F3B—C8—C7	98.8 (7)
C3—N3—Cu1	109.77 (15)	F2A—C8—C7	114.9 (4)
C3—N3—H3A	109.7	F3A—C8—C7	115.3 (7)
Cu1—N3—H3A	109.7	F2B—C8—C7	113.1 (5)
C3—N3—H3B	109.7	F1A—C8—C7	111.6 (7)
Cu1—N3—H3B	109.7	O4—C9—O3	128.0 (3)
H3A—N3—H3B	108.2	O4—C9—C10	114.6 (3)
N3—C3—C4	110.8 (2)	O3—C9—C10	117.4 (3)
N3—C3—H3C	109.5	F6A—C10—F5A	109.5 (6)
C4—C3—H3C	109.5	F6A—C10—F4B	79.6 (10)
N3—C3—H3D	109.5	F5A—C10—F4B	117.5 (7)
C4—C3—H3D	109.5	F6A—C10—F5B	135.3 (9)
H3C—C3—H3D	108.1	F5A—C10—F5B	28.1 (8)
N4—C4—C3	110.08 (19)	F4B—C10—F5B	105.9 (4)
N4—C4—H4C	109.6	F6A—C10—F6B	34.5 (11)
C3—C4—H4C	109.6	F5A—C10—F6B	77.3 (8)
N4—C4—H4D	109.6	F4B—C10—F6B	105.2 (4)
C3—C4—H4D	109.6	F5B—C10—F6B	105.2 (3)
H4C—C4—H4D	108.2	F6A—C10—F4A	105.5 (7)
C4—N4—Cu1	105.13 (14)	F5A—C10—F4A	106.4 (8)
C4—N4—H4A	110.7	F4B—C10—F4A	26.0 (9)
Cu1—N4—H4A	110.7	F5B—C10—F4A	86.2 (7)
C4—N4—H4B	110.7	F6B—C10—F4A	128.0 (12)
Cu1—N4—H4B	110.7	F6A—C10—C9	105.3 (6)
H4A—N4—H4B	108.8	F5A—C10—C9	120.7 (7)
C5—N5—Cu1	109.29 (14)	F4B—C10—C9	114.9 (4)
C5—N5—H5A	109.8	F5B—C10—C9	111.5 (4)
Cu1—N5—H5A	109.8	F6B—C10—C9	113.3 (3)
C5—N5—H5B	109.8	F4A—C10—C9	108.5 (9)
Cu1—N5—H5B	109.8		

### Hydrogen-bond geometry (Å, °)

**Table:**

*D*—H···*A*	*D*—H	H···*A*	*D*···*A*	*D*—H···*A*
N2—H2B···F1A	0.90	2.54	3.248 (17)	136
N2—H2B···F3B	0.90	2.55	3.41 (2)	161
N6—H6B···O1	0.90	2.15	3.048 (3)	179
N1—H1A···O2^i^	0.90	2.35	3.141 (3)	147
N1—H1A···O1^i^	0.90	2.53	3.377 (4)	156
N4—H4A···O2^i^	0.90	2.34	3.175 (3)	154
N6—H6A···O1^i^	0.90	2.56	3.326 (3)	143
N1—H1B···O2^ii^	0.90	2.11	3.002 (3)	173
N2—H2A···F4A^ii^	0.90	2.47	3.267 (15)	148
N3—H3A···O3^ii^	0.90	2.09	2.958 (3)	162
N5—H5A···O3^ii^	0.90	2.36	3.126 (3)	143
N2—H2A···O4^iii^	0.90	2.41	3.037 (3)	127
N4—H4B···O4^iv^	0.90	2.11	2.997 (3)	168
N5—H5B···O3^iv^	0.90	2.13	3.013 (3)	167

Symmetry codes: (i) −*x*+2, −*y*+1, −*z*+2; (ii) *x*, *y*+1, *z*; (iii) −*x*+1, −*y*+1, −*z*+1; (iv) −*x*+2, −*y*+1, −*z*+1.
